# An Ex Vivo Animal Organ Model Tailored for Fluoroscopically Guided Percutaneous Jejunostomy

**DOI:** 10.1007/s00270-025-04022-w

**Published:** 2025-04-15

**Authors:** Xiang Geng, Rui-Can Nie, Yang Zhao, Hai-Liang Li, Hang Yuan, Hong-Tao Cheng, Shi-Jun Xu, Ya-Na Ma, Dong-Yang Zhang, Yao Chen, Hong-Tao Hu, Ho-Young Song

**Affiliations:** Department of Interventional Radiology, The Affiliated Cancer Hospital of Zhengzhou University/Henan Cancer Hospital, NO. 127, Dongming Road, Zhengzhou, 450003 Henan Province China

To the Editor,

For patients with severe gastroparesis, previous gastrectomy, or gastric outlet obstruction, percutaneous jejunostomy is a valuable therapeutic option for providing enteral nutrition [1, 2]. Although percutaneous jejunostomy is performed worldwide, it remains a technically demanding procedure with a steep learning curve and relatively high failure rates [2].

To enhance physicians’ proficiency in complex surgical techniques or novel procedures, various training tools have been employed, including animal models [3], cadavers, and simulators [4].

This letter primarily introduced an ex vivo animal organ model we developed, tailored for fluoroscopically guided percutaneous jejunostomy (Figs. 1 and 2). The model was designed to help novice physicians familiarize themselves with this complex procedure before performing it on animal models or patients.

**Fig. 1:**
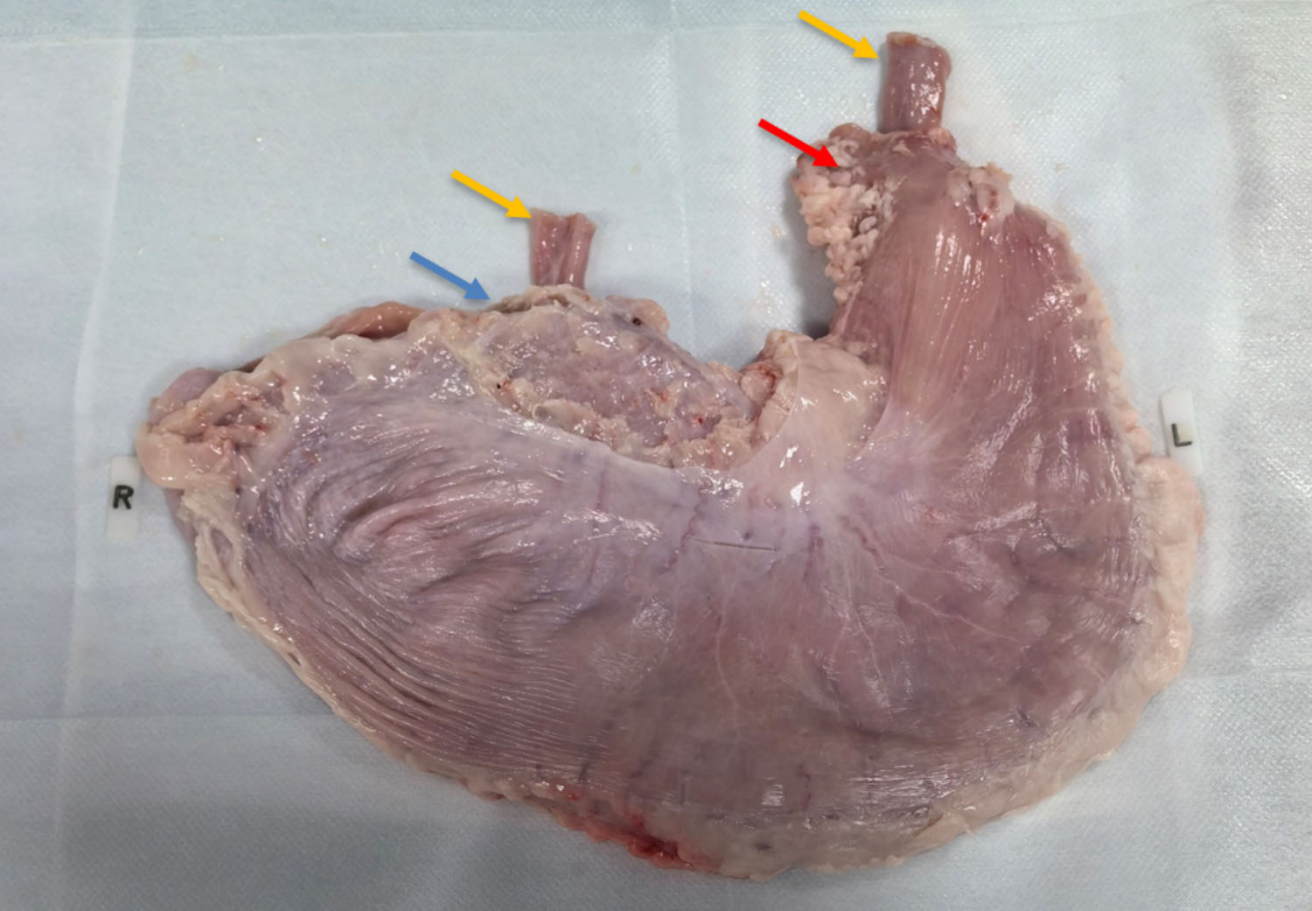
In vitro animal organ model for fluoroscopically guided percutaneous jejunostomy. The red arrow indicates the cardiac part of the adult pig’s stomach, the blue arrow indicates the pylorus, and the yellow arrow indicates the end of the jejunum

**Fig. 2:**
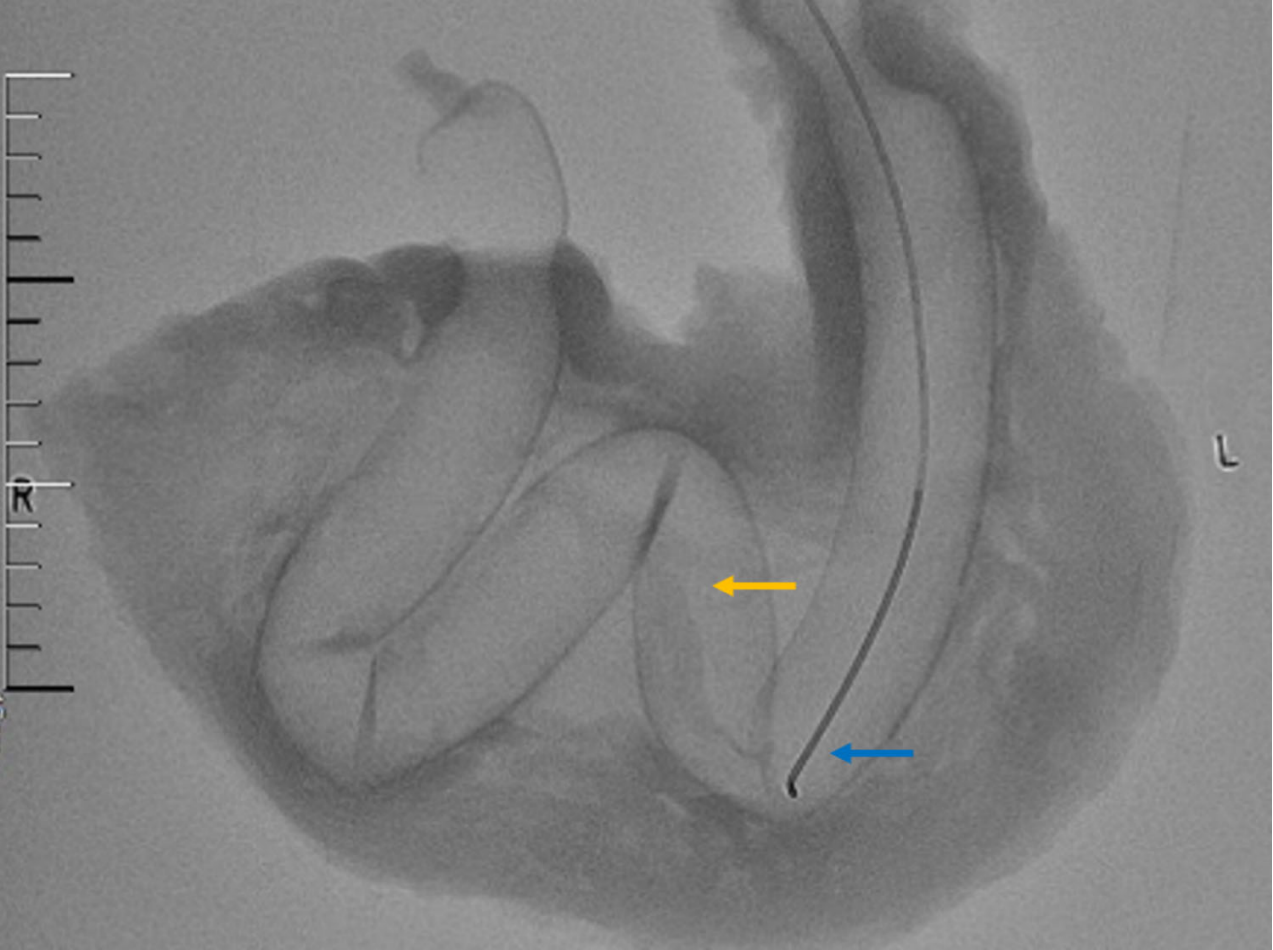
Fluoroscopically guided percutaneous jejunostomy: A 5F single-curved catheter (blue arrow) has been inserted into the jejunum, and air has been injected. The yellow arrow indicates the inflated jejunum

## Fabrication of the Ex Vivo Animal Organ Model

Fresh stomach and jejunum from healthy adult porcine specimens were purchased from a local slaughterhouse on the day of the experiment. A 10-cm incision was made along the greater curvature of the stomach to clean the gastric cavity and facilitate jejunum placement. Blood and debris were removed by rinsing with clean water, followed by washing the mucosal surface with normal saline. The length and thickness of porcine stomachs were measured and recorded. The organs were then wrapped in gauze soaked in normal saline, placed in a refrigerated preservation box at 4 °C, and immediately brought to the laboratory.

A 70-cm segment of porcine jejunum was inserted into the gastric cavity through the greater curvature of the stomach. One end of the jejunum was pulled out through the cardia, while the other end was extracted through the pylorus and ligated with silk thread. Under fluoroscopic guidance, a vertebral catheter (Terumo, Japan, 5F) was inserted into the jejunum at the cardia and advanced to the distal end at the pylorus. The jejunal opening at the cardia was then ligated with silk thread, ensuring the catheter remained patent. Subsequently, approximately 30 mL of air was injected using a 10-mL syringe to slightly distend the jejunum, which was then neatly positioned inside the gastric cavity. The incision along the greater curvature was sutured with silk thread, followed by insufflation of air into the jejunum to achieve optimal distention.

## Fluoroscopically Percutaneous Jejunostomy Procedure Simulation

**Fig. 3:**
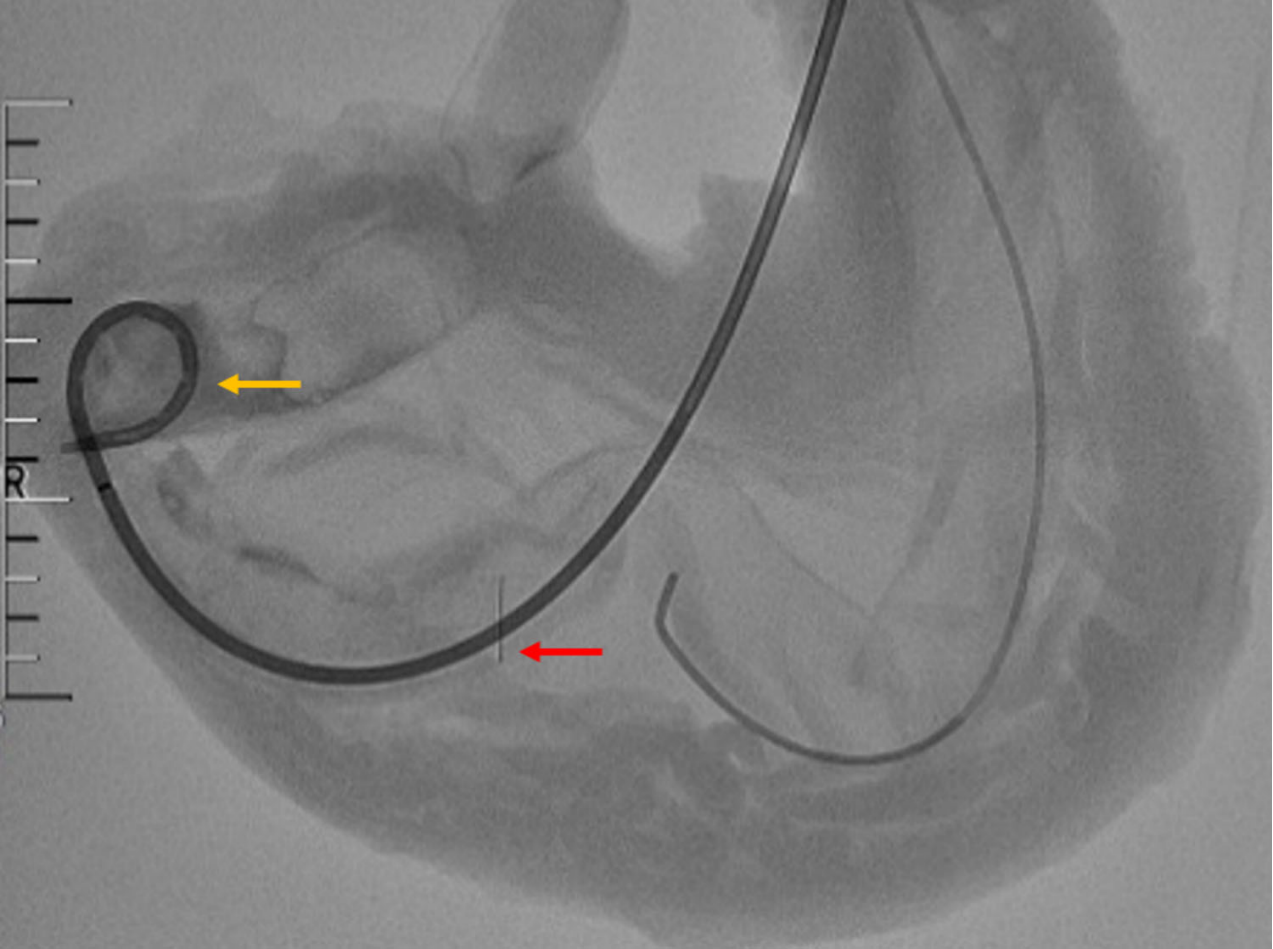
Fluoroscopically guided percutaneous jejunostomy: The drainage tube has been fixed in a loop (yellow arrow) and its position has been determined by contrast. The red arrow indicates the anchor

The model creation process involved the following steps: Under fluoroscopic guidance, a puncture point located in the middle of the stomach wall was selected. A 21-gauge puncture needle (Merit Medical, USA) was then used to puncture both the gastric wall and the anterior wall of the jejunum at the designated point. Following successful puncture, a 0.018-inch guidewire (Merit Medical, USA) was inserted into the jejunum through the 21-gauge needle. The guidewire was retained while the needle was withdrawn. A 6F sheath (Merit Medical, USA) was then advanced over the guidewire into the jejunum, and its position was confirmed by imaging. Subsequently, an anchor (self-made, the diameter is 0.018 inches) was inserted through the 6F sheath into the jejunum, followed by the insertion of a 0.035-inch guidewire (Terumo, Japan) through the 6F sheath while retaining both the anchor and guidewire. The 6F sheath was withdrawn, and the anchor was pulled to appose the jejunal wall to the gastric wall. Finally, a 10-F drainage tube (EVD Medical, China) was inserted into the jejunal lumen over the guidewire, and the tube was looped (Fig. 3).

This ex vivo model does not fully replicate clinical conditions, as neither glucagon nor the amount of air typically used in percutaneous jejunostomy procedures in patients or in vivo studies were applied.
